# Health Related Quality of Life May Increase when Patients with a Stoma Attend Patient Education – A Case-Control Study

**DOI:** 10.1371/journal.pone.0090354

**Published:** 2014-03-07

**Authors:** Anne Kjaergaard Danielsen, Jacob Rosenberg

**Affiliations:** Department of Surgery, Herlev Hospital, University of Copenhagen, Copenhagen, Denmark; University of British Columbia, Canada

## Abstract

**Introduction:**

Adaptation to living with a stoma is complex, and studies have shown that stoma creation has a great impact on patients' health related quality of life. The objective was to explore the effect of a structured patient education program on health related quality of life. Therefore, we implemented interventions aimed at increasing health related quality of life during and after hospital admission.

**Materials and Methods:**

We designed a case/control study aimed at adult patients admitted to the surgical ward for stoma creation, irrespective of type of stoma or reason for creation of stoma. We included 50 patients in the study. Health related quality of life was measured before hospital discharge, three months and six months after stoma creation. The program included educational interventions involving lay-teachers, alongside health professional teachers.

**Results:**

We found a significant rise in health related quality of life in the intervention group (P<0.001) and no significant change in the control group (P = 0.144). However, we found no significant differences when comparing between groups at 3 and 6 months (*p* = 0.12 and *p* =  0.63, respective). Additionally, there were differences in scores in health related quality of life baseline (*p = 0.045*) with lower scores in the intervention group compared with the intervention group. However, there were no significant differences in the demographic variables at baseline

**Conclusions:**

Educational activities aimed at increase in knowledge and focusing on patients' psychosocial needs may lead to a rise in patients' health related quality of life. When patients with a stoma attend a structured patient education program it is possible to improve their health related quality of life compared with patients with a stoma, who do not attend the program.

**Trial Registration:**

ClinicalTrials.gov NCT01154725

## Introduction

Stoma creation affects patients differently [1], but generally we know that health related quality of life is impaired when trying to adjust to a life with a stoma [2], [3]. Stomas are constructed for different reasons, typically as treatment for cancer, or after trauma, or because of inflammatory bowel disease.

A previous study has shown that appropriate patient education in the preoperative period may reduce time until proficiency in stoma handling, as well as time until discharge from hospital [4]. Furthermore, there is some evidence showing, that patient education may help to increase patients' knowledge about their health, their condition and their self-care possibilities [5] Few studies have explored the issue of the effect of patient education on health related quality of life, and only in single-group studies [6], [7]. Moreover, a recent systematic review examining the effect of patient education in patients with a stoma concluded that there was a need for testing the effect in a more controlled design [8].

Therefore, we designed a clinical study exploring the effect of structured patient education in patients after stoma creation. We hypothesized that patient education and telephone follow-up would affect treatment outcome of health related quality of life (hrqol).

## Materials and Methods

We designed a case-control study including patients admitted to the surgical department for stoma creation. Patients were included from August 2010 until June 2011 with a follow-up period of 6 months after surgery. The inclusion was sequential. First we included and studied the control group receiving routine stoma care, and subsequently the intervention was implemented and the intervention group was included.

We obtained an informed oral and written consent from all participants included in this study. The protocol for this trail and supporting CONSORT checklist are available as supporting information; see Checklist S1 and Protocol S1.

### Outcomes

The main objective of the study was to explore whether the interventions would improve hrqol 6 months after stoma creation. We applied the questionnaires Ostomy Adjustment Scale (OAS) and Short Form 36 (SF-36), and patients were scored three times: a few days before discharge, and three months and six months after discharge.

Furthermore, we explored the costs related to the implementation of the patient education program, and the results are reported elsewhere [9].

### Sample size

The primary outcome was hrqol measured by OAS 6 months after stoma creation. In the literature we found no data for OAS measured 6 months after surgery. However, there were data describing means and standard deviations for outcome measured from 1 to 30 years after surgery [10], [11]. With this in mind we set the minimal relevant difference at 15% with a mean OAS score at 155 points, SD at 23, type I error at 5%, and a type II error at 20%. In a 2 sided-test the necessary number of patients would be 16 in each group. When accounting for drop-outs we chose to include 50 patients.

### Inclusion criteria

We included adult patients (18 years+) admitted to the surgical department after stoma creation no matter whether the stoma was a colostomy or an ileostomy, irrespective of whether it was expected to be permanent or temporary, and regardless of reason for creation of the stoma.

### Exclusion criteria

Patients, who withdrew their consent to participate.

### Usual stoma care which was provided for the control group

Patients admitted to the surgical department for a planned stoma creation received preoperative education by the stoma therapist. Patients operated on an acute basis did not meet the enterostoma therapist (ET) before 4–5 days after stoma creation, just before leaving hospital. The preoperative education in the elective setting included information on stoma care, and marking of the stoma site on the patient's abdomen. After surgery, the patient would be guided by the nurses in the surgical ward regarding stoma care. The ET had a discharge dialogue with the patient before leaving hospital, and once at home, the patient had scheduled contacts with the ET at 10 days, 1 month, 3 months, 6 months, and 12 months after stoma creation. Furthermore, patients would have access to contact with the out-patient clinic by telephone.

### Interventions only set up for the intervention group

The intervention group received the same interventions as the control group. Additionally, patients in the intervention group were visited in the ward by the ET two (+/- 1) days after stoma creation. The visit was set up to guide and supervise the patient when changing the pouch, often for the first time. Furthermore, the ET would prepare a plan for the clinical care of the patient to guide the patient as well as the ward nurses. The visit was guided by a checklist developed by the first author.

Furthermore, the ET contacted the patient by telephone 5 (+/- 2) days after discharge, and assessed the patient's well-being following a checklist. Questions related to stoma care were resolved, and any need for face-to-face visits at the stoma clinic was examined by the ET.

Patient education sessions were set up after hospital discharge and were organized according to issues that were identified as relevant and central for patients after stoma creation [12], [13]. The educational approach was inspired by theories of self-management, and self-efficacy, and was based on principles of adult learning and health education [14]. The sessions were conducted in groups with an upper limit of 8 participants, and an ET would follow the group as a course director (table 1). All sessions lasted 3 hours and were held at the hospital, and involved physiotherapists and a sexologist, who all had extensive experience with this specific group of patients. Furthermore, we invited a lay teacher (who had a stoma herself), recruited with the assistance from the Danish Ostomy Association COPA. The involved teachers as well as the course directors were all instructed by the first author, who also supervised sessions.

**Table 1:** Overview of the educational sessions.

	Theme	Teacher
**Session 1** (between 4 weeks and 8 weeks after stoma creation)	**Everyday life with an enterostoma** Common problems: gas, odor, leaks Changing the pouch Family and friends What to eat and drink after stoma creation Clothing	Stoma therapist
	**Experiences with living with an enterostoma** Travelling Going to work Living in a family	Lay teacher from patient organization for persons with a stoma in Denmark
**Session 2** (between 8 weeks and 16 weeks after stoma creation)	**Skin care and special appliances** Dermatitis, hyperplasia Constipation, diarrhea Scars, parastomal bulging	Stoma therapist
	**Exercises and training after stoma creation** Benefits and risks related to exercising Fitness, bicycling, jogging, swimming Exercising with others Instruction and supervision	Physiotherapist
**Session 3** (between 16 weeks and 24 weeks after stoma creation)	**Back to work, social and physical activities** How to resume activities from life-before-the stoma Coherence between life before and after stoma creation	Stoma therapist
	**Sexuality and intimacy** Common sexual problems related to colorectal surgery and stoma creation How to gain or regain intimacy and intercourse Body-image and identity Treatment options for sexual complications	Sexologist

### Health related quality of life

Hrqol was assessed before leaving hospital, 3, and 6 months after stoma creation. As a disease-specific quality of life measurement the Ostomy Adjustment Scale (OAS) was used to assess the disease-specific adjustments [10], [11]. The questionnaire was designed to measure patients' reactions to an incontinent stoma and the adjustment to living with a stoma. The level of adjustment was related to physical, psychological and social changes that might occur after stoma creation, and was defined as the patient's subjective response to having a stoma. The questionnaire consisted of 34 questions, and had been tested for reliability and validity, and was translated into Danish in a forward-backwards process as well as face validation [15]. Each item was scored from 1 to 6 (worse to better adjustment) with possible scores ranging from 34 to 204.

Short form 36 v2 was a generic tool evaluating quality of life from patients' self-reports on different conditions influencing quality of life [16] and had been validated for use in a Danish context [17]. It consisted of 36 items measuring 8 dimensions of health on a multi-item scale, with scores ranging from 0 to 100 (with lower scores indicating worse health). The eight multi-item scales were: physical functioning (PF), role limitations-physical (RP), bodily pain (BP), general health (GH), vitality (VT), social functioning (SF), role limitations-emotional (RE), and mental health (MH). Originally we aimed at applying another questionnaire measuring generic health related quality of life (EQ-5D), but refrained from this as we believed that SF-36 would be more sensitive to changes in scores in our participants.

### Data analysis

Data analysis was based on descriptive statistics and nonparametric tests using IBM SPSS statistics version 20. Descriptive data were reported as median with range. Comparisons between or within groups or between groups were made using Fisher's exact test, Friedmańs test, and Mann Whitney test where applicable. Statistical significance was set at *p*≤0.05. Furthermore, we performed a missing data analysis using binary logistic regression. The plan for analysis was based on an intention-to-treat strategy.

### Ethics

Approval of the study was obtained from the Danish data protection agency (J.nr. 2010-41-4706). The study was performed in compliance with the ethical principles of the World Medical Association declaration of Helsinki. However, the Danish Regional committee evaluated that the study was exempt from approval because we aimed at quality assurance rather than performing a biomedical intervention (H-2-2010-041). Furthermore, the study was notified on www.clinicaltrials.gov (NCT01154725).

## Results

**Figure 1:**
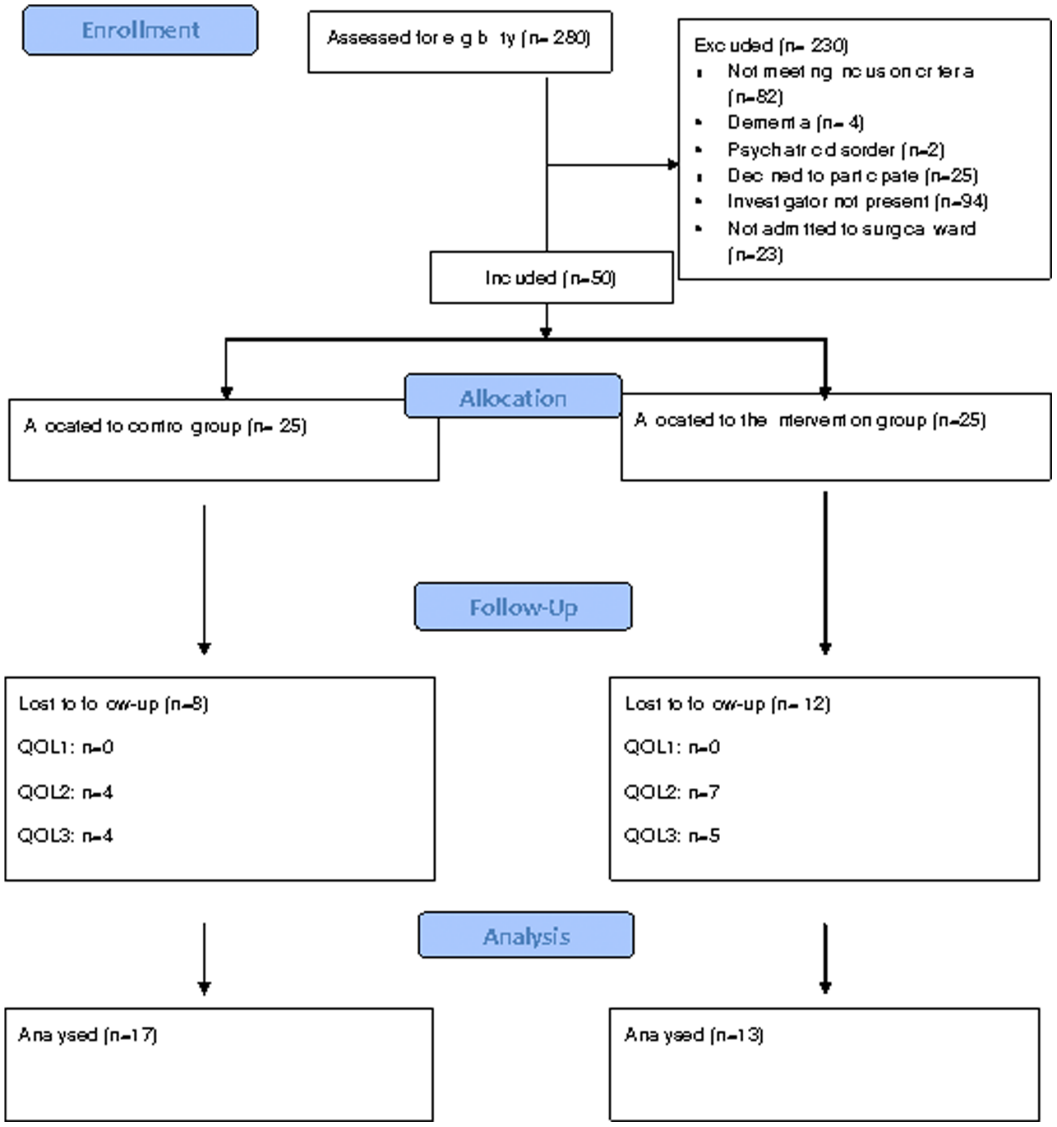
Consort 2010 Flow diagram.

Of the 280 eligible participants 75 were invited to participate. 25 of these declined and we included 25 patients in each group (figure 1). After the inclusion of 25 patients in the control group the intervention was implemented, and further 25 patients were included. Baseline demographic characteristics of the patients showed no significant differences between groups (*p*<0.05, table 2). A Fisher's exact test revealed no significant difference between drop-out rates in the control group and the intervention group (*p* = 0.38 at the third visit, 6 months after stoma creation). Furthermore, we performed a binary logistic regression analysis to explore whether gender, cancer/non-cancer, and outcome were significantly related to the dropout of participants, showing no difference between dropouts and patients still in the study (p = 0.19, p = 0.70, p = 0.90, respectively). We did not use an intention-to-treat analysis as we experienced missing of data. However, all participants were analyzed within the group to which they were originally allocated.

**Table 2:** Baseline characteristics of participants with percentages in brackets.

	Control group (n = 25)	Experimental group (n = 25)
Age (median (range))	65(30–83)	67(49–80)
Gender (male/female)	13(52)/12(48)	10(40)/15(60)
Colostomy/ileostomy	12(48)/13(52)	19(76)/6(24)
Permanent/temporary	17(68)/8(32)	19(76)/6(24)
**Reason for stoma creation**		
Cancer	16(64)	18(72)
IBD	6(24)	4(16)
Other	3(12)	3(12)
**Comorbidity**		
Ischaemic heart disease	2(8)	2(8)
Hypertension	12(48)	5(20)
COPD	0(0)	1(4)
Diabetes	0(0)	0(0)
Renal insufficiency	0(0)	0(0)
Other	1(4)	0(0)
**Lifestyle**		
Smoking	3(12)	2(8)
Use of alcohol (>60 g alcohol/day)	1(4)	0(0)
**Acute surgery**	2(8)	8(32)

There were no significant differences between groups.

IBD  = Inflammatory bowel disease, COPD  =  Chronic obstructive pulmonary disease.

### Health related quality of life

#### OAS

**Figure 2:**
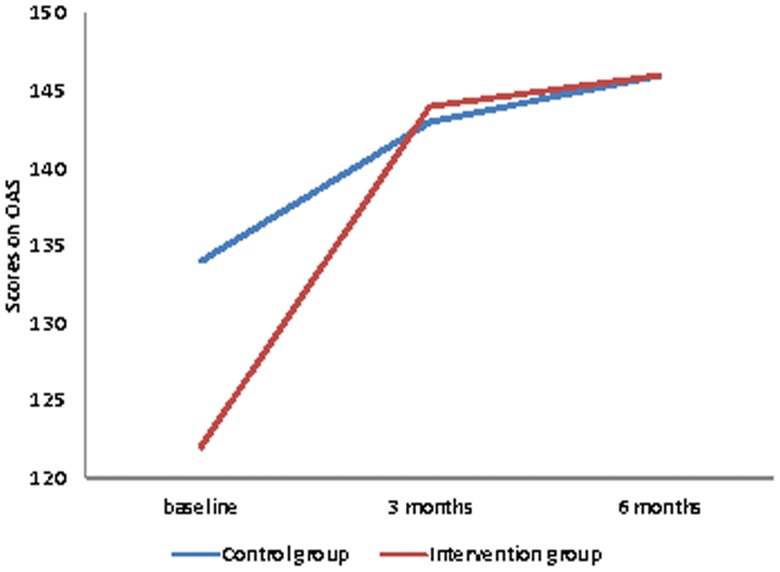
Scores on Ostomy Adjustment Scale (OAS). Data are median values for the group. Footnote: Presenting scores with (P<0.001) or without (P = 0.144) educational intervention. Data shown are from the participants, who were present at all three points of measurement.

The intervention group showed a significant development in OAS scores between baseline, three months and 6 months (Friedman test, p<0.001, figure 2). In the control group there was no statistically significant variation in the OAS scores throughout the study period (Friedman test, *p* = 0.14, figure 2). However, there were baseline differences (*p = 0.045*) between the groups with lower scores in the intervention group (score 122) compared with the intervention group (score 134). Moreover, there were no significant differences between groups 3 and 6 months after stoma creation (*p* = 0.12 and *p* =  0.63, respective).

#### SF36

We found significant changes in the control group in physical functioning (P = 0.001), bodily pain (*p* = 0.003), and mental health (*p* = 0.02). In the intervention group there were significant changes in bodily pain (*p* = 0.004) and mental health (*p* = 0.04). None of the other dimensions of health showed significant variation throughout the study period (table 3). There were no differences between groups in the profile scores at baseline, 3 and 6 months after stoma creation (*p* ranging from 0.93 to 0.11, Mann-Whitney test).

**Table 3:** SF-36v2 scores. Values are median (range).

	Baseline(n = 25)	3 months(n = 21)	6-months(n = 17)	*P*
**Control group**				
PF	52 (5—95)	74 (10–95)	80 (25–100)	0.001
RP	43 (0—100)	45 (0–100)	52 (0–100)	ns
BP	46 (0–100)	82 (22–100)	85 (52–100)	0.003
GH	68 (10–97)	71 (20–92)	67 (27–87)	ns
VT	50 (0–93)	61 (12–100)	63 (0–100)	ns
SF	71 (12–100)	84 (37–100)	80 (0–100)	ns
RE	67 (0–100)	63 (0–100)	65 (0–100)	ns
MH	62 (10–100)	74 (25–100)	74 (10–100)	0.03
**Experimental group**	(n = 25)	(n = 18)	(n = 13)	
PF	56 (5–100)	65 (5–95)	66 (0–100)	ns
RP	44 (0–100)	51 (0–100)	58 (6–100)	ns
BP	51 (10–100)	71 (22–100)	77 (22–100)	0.004
GH	63 (16–100)	68 (45–92)	72 (42–92)	ns
VT	42 (0–93)	52 (6–81)	55 (6–81)	ns
SF	59 (25–100)	72 (25–100)	75 (25–100)	ns
RE	63 (0–100)	68 (0–100)	71 (25–100)	ns
MH	56 (5–100)	65 (10–95)	75 (40–100)	0.04

PF = physical functioning, RP = role physical, BP =  bodily pain, GH =  general health, VT =  vitality, SF =  social functioning, RE =  role emotional, MH =  mental health. Only showing significant *p* values (Friedman analyses) ns = not significant.

## Discussion

We explored whether establishment of patient education activities and additional support from the ET would increase hrqol in patients after stoma creation. The analysis showed that patients allocated to a patient school program aimed at rehabilitation had a significant improvement in hrqol measured by OAS with no changes in the control group. There was a significant difference between groups at baseline, though. When using a generic quality of life questionnaire (SF-36) the improvements were only significant in some of the profiles. However, there were no differences between groups in OAS or SF-36 at 6 months after stoma creation.

The differences at baseline in scores on OAS might be because of a systematic inclusion bias, where we have included patients in the intervention group, who were characterized by lower hrqol scores. When looking at the demographic data at baseline, we were not able to detect significant differences (table 2), nor were we able to identify any differences related to gender or underlying disease when looking at patients dropping out of the study. As such, the significant changes in hrqol scores, which were only found in the intervention group, were most likely because of a beneficial effect of participation in the educational program. On the other hand, it should be discussed whether the positive results in the intervention group indicated that the intervention would primarily affect patients with lower scores. Furthermore, because of drop outs there were fewer patients in the study at 6 months than the power calculation demanded (13 patients in the intervention group as opposed to the demanded 16 patients). As such, the results were not as strong as desired in order to make clear conclusions about the outcome.

Previous studies have explored the impact of patient educational activities directed towards patients with a stoma [4], [6]–[7], [18]–[19]. However, only few have designed studies involving a control group [4], [18]–[19]. One of these studies showed that preoperative patient education in the patients' home had a significant effect on the patients gaining proficiency in stoma management [4]. However, due to logistic problems we were unable to include this intervention in our study. The other two, were Taiwanese studies exploring the effect of a multimedia education program for patients with stomas, with a focus on knowledge increase [18] and cost effectiveness [19] and found significant effects. However, the results were difficult to transfer to a Danish clinical context as the interventions applied seemed less intensive than the standard patient course in Denmark.

However, the results of our study might add to the results of two interventional single-group studies aimed at exploring the effect of patient education on health related quality of life [6], [7]. Our interventions were alike, but not having a control group made it difficult to rule out that the increases in hrqol in the two studies were more than coincidental.

Other studies have suggested that patient education related to patients with a stoma should focus more on socially oriented elements [20] and self-management [21]. This might indicate that patient education should also focus on concerns and questions that are less plain and evident, and maybe involving lay teachers with a stoma may support this [12], [13].

The differences when using different questionnaires probably reflected that the OAS, being a disease-specific questionnaire, was more sensitive to the stoma related problems that the patients perceived. SF 36 is a generic questionnaire and was obviously not as sensitive to the changes and the feelings experienced by the patients in our study.

The results of this study may have been influenced by the drop-out rate and therefore a loss of data. Furthermore, the inclusion rate was low, as the researcher did the inclusion on her own, and is a well known difficulty when doing clinical research [22]. Additionally, we did not design a randomized controlled trial, as we expected that patients in the intervention group might have a substantial confounding influence on patients in the control group. The random allocation of patients would have been a superior design, and we discussed whether we could have implemented the study in two separate settings. This would have made it possible for us to perform parallel inclusion processes, shifting the randomization from a patient level to a hospital level. However, this design would have resulted in even more confounding variables connected to different nurses and surgeons, as well as differences in the standard course. Therefore, we decided that a case-control study with sequential inclusion would be the most fitting design.

In conclusion, we have shown that establishment of a structured patient education program aimed at patients with a stoma improved disease specific areas within health related quality of life. A program including interventions aimed at increasing knowledge as well as self-management might benefit from including lay-teachers alongside health professional teachers. Furthermore, the use of telephone follow-up after discharge from hospital may increase patients' health related quality of life.

## Supporting Information

Protocol S1 Trial Protocol.
(PDF)

Checklist S1 CONSORT Checklist.
(PDF)
