# Supplementary material for: Health Related Quality of Life May Increase when Patients with a Stoma Attend Patient Education – A Case-Control Study
Source: PLoS One. 2014 Mar 7;9(3):e90354. doi: 10.1371/journal.pone.0090354 (PMC3946520; doi:10.1371/journal.pone.0090354)
Supplement: Protocol S1 — Trial Protocol. (PDF) [file pone.0090354.s001.pdf]

Study protocol version 4 22-03-2010

The effect of patient education and rehabilitation on quality of life in patients with a stoma

Contact:
Anne Kjaergaard Danielsen
RN, MClN, Ma(ed)
Department of Surgery,
Herlev Hospital, University of Copenhagen
Tel : 44884000 dial tone 82412
email: adan0009@heh.regionh.dk

Objectives of the study:

Primary outcome:

1 The objective of this study was to explore the effect of patient education on health related quality of life 6 months after stoma creation.

Secondary outcome:.

2. An analysis of the cost effectiveness based on the results of the primary outcome.

Background

The impact of stoma creation on the individual is characterized by a variety of factors that will impact the individual: several types of stoma, differences in the underlying causes, and individual psychosocial factors (1-4). Patients must adjust to a changed body image, changing daily routines and for some there will also be a change in lifestyle (5), and affected sexuality (6).

Moreover, personal factors do probably affect individual´s adaptation to life with a stoma; including age (7), socioeconomic status (8) and gender (9). The patient course for patients with a stoma may be perceived as disruptive when the patient is being discharged from hospital. As such, patients may experience that the intense practical and emotional support connected to the hospital environment may be replaced by a requirement to take care of themselves at home (10-12) .

Since the training of the first enterostoma therapist (ET) in the 1960´s, the professional activities have been centered on training and instruction of patients. These tasks are increasingly being carried out by ET´s both in Denmark and internationally (13).

The primary role of the ET is to inform and prepare patients and possibly relatives preoperatively and maintain contact with patients post-operatively in a rehabilitative process (12, 14-17). It is assumed that the interventions and involvement by the ET is important for the patient's quality of life (qol), and one study concluded that training by an ET had a positive impact on quality of life compared to no training (18). Furthermore, a study concluded that preoperative training in the home of the patient had an effect on length of stay after surgery (19) However, a literature review (20) only found limited evidence that the involvement of an ET had an independent and positive effect on patients' qol. Nevertheless, other studies suggested that patients who are satisfied with the ET had a higher score in health-related quality of life (21) and the patients seem to point at the possibility of guidance, support and advice to be of great importance (2). However, there are no studies that conclusively identified the most effective way to implement patient education, neither regarding content or didactical methods (22,23).

Teaching of surgical patients seemed to have a positive impact on patients' quality of life (24) and a review indicated that preoperative education of patients had a positive impact (25). It has been suggested that interventions where the focus of the intervention is the individual needs of the may have the greatest impact

(22,26,27): Moreover, a study pointed at telephone follow-up after discharge from hospital had a favorable effect on health-related quality of life (HRQOL) in patients after hip replacement surgery (28).
Additionally, the measurement of HRQOL is increasingly demanded in the health care (29,30), and seems to be a need for testing the effect of nursing interventions on patients' HRQOL (31). Furthermore, as the resources in the health care sector are limited, it may be fruitful to explore other tools than the strictly health scientific, and move on to assessing the health economic effects when establishing a patient education program aimed at patients with a stoma (32,33).

Hypotheses

primary:

Patients with a stoma will experience an improvement in health-related quality of life associated with the implementation of a patient education program.

secondary:

Cost analysis will show that the introduction of patient school is cost-effective compared to the standard course.

Inclusion:

Adult patients admitted for elective or acute stoma creation irrespective of reason for the stoma creation.
Patients who are expected to take care of stoma patients themselves.
Patients, who can cooperate with completion of questionnaires and use of diary.
Patients will be included consecutively after stoma creation.

Exclusion :

Patients, who postoperatively cannot attend the course as a result of physical, mental or social constraints
Patients, who withdraw their consent of participation.

Outcome measures :

Primary outcome:

Scores on the questionnaires Ostomy Adjustment Scale (OAS) and EQ-5D will be assessed and compared six months after discharge.

Secondary:

Registration and comparison of costs for the control and intervention group.

Design:

The study will be conducted as a case-control study in which the interventions will be implemented after inclusion of the control group. The study group will be included following the implementation of the interventions. This design is preferred in order to avoid confounding, as the intervention cannot be blinded to either personnel or patients.

Sample size

The primary outcome was hrqol measured by OAS 6 months after stoma creation. In the literature we found no data for OAS measured 6 months after surgery. However, there were data describing means and standard deviations for outcome measured from 1 to 30 years after surgery (9,10). With this in mind we set the minimal relevant difference at 15% with a mean OAS score at 155 points, SD at 23, type I error at 5%, and a type II error at 20%. In a 2 sided-test the necessary number of patients would be 16 in each group. When accounting for drop-outs we chose to include 50 patients.

Description of methods:

Quality of life:

Health related quality of life will be measured in both groups.

To measure and describe the disease specific quality of life of stoma we will use the questionnaire Ostomy Adjustment Scale (OAS). Furthermore, we will measure and compare generic health-related quality of life with EQ-5D.

Ostomy Adjustment Scale (OAS, disease-specific quality of life) and EQ-5D (generic quality of life) are both designed for either completion in the presence of the interviewer, self-completion, or telephone completion.

The studies carried out by self-completion either alone or in the presence of the interviewer.

Registration of data related to costs:

Data will be registered in both groups.

During hospitalization: Demographic data: gender, age , ethnicity, whether they live alone , reliance by others in everyday life, education, and current position.

From the electronic journal system: surgical technique, admittance to hospital.

Furthermore, we will access the registry of the hospital, and a diary provided to the patient. Data related to the hospital course will cover: length of stay, readmission to hospital, use of out-patient clinic.

Costs related to primary care will include visits with the general practitioner, contact to primary nurse, and physical and social activities.

Patient education program

The patient education programme will only be conducted in patients in the study group, and patients in the control group will have standard care.

Structured interviews day 1(+/- 2 days) postoperatively will be conducted by the ET with a perspective of health promotion addressing specific stoma related issues.

5 days (+/- 2 days) after discharge from hospital the ET will contact the patient by telephone, and guide the patient on the basis of a check list.

Patient education session will be performed with an extent of 3 afternoon sessions along with 4-6 other patients. Sessions will be focusing on individual and collective processes, and with a content which targets the issues of a general interest to people after stoma creation. The issues will be everyday life with a stoma, life in the family, to return to work / social communities, sexuality and problems in connection with disease and ostomy particular. Teaching is carried out by healthcare professional and the course directors (ET´s).

Risks, adverse effects and problems associated with the study

We have no knowledge of adverse effects and risks associated with participation in this type of rehabilitative process. However, attention will be paid to patients' experiences especially during the group processes. If any of the participants need to be seen by a physician this will be handled by the course directors, who all are registered and experienced nurses.

Measurement of quality of life by completing questionnaires before surgery and 3 and 6 months after surgery is not expected to result in either adverse effects or risks for the patient.

Ethical considerations

Patients included in the project will be informed thoroughly both in writing and orally. The information covers the planned course during hospitalization and the follow-up period of 6 months. Patients will be able to have a family member present during the information session, and the voluntary participation will be emphasized, as well as the possibility of withdrawal of consent.

Guidelines for informed consent

The researcher provides the oral information to patients prior to inclusion, which will place in the ward. The interview will be held an undisturbed place, either in the interview room or if the patient is bedridden ensures that the patient agrees to be informed in the hospital ward. Fellow patients will be asked to leave the room, if possible. Patients are encouraged to have a caregiver present at the information interview. Time for consideration will be short, and up to 24 hours.

Notification to the Data Protection Agency

The project requires ethical review.

Financial support

The project is initiated in the research group.

Payment of included participants

The included participants will not receive any payment, but transport costs will be refunded.

Publication of results

All results both negative and positive will be published.

Reference List

(1) Brown H, Randle J. Living with a stoma : a review of the literature. J Clin Nurs January 2005 , 14:74 - 81

(2) Persson E , Wilde LB . Quality of care after ostomy surgery : a perspective study of patients. Ostomy Wound Manage 2005; 51:40-8

(3) Persson E , Gustavsson R, Hellstroem A, Lappas G , Hulten L. Ostomy patients' perceptions of quality of care. Journal of Advanced Nursing 2005;49:51-8

(4) Nugent KP , Daniels P , Stewart B , Patankar R , Johnson CD. Quality of life in stoma patients. Dis Colon Rectum 1999;42:1569-74

(5) Gooszen AW, Geelkerken RH, Hermans J, Lagaay MB, Gooszen HG. Quality of life with a temporary stoma: ileostomy vs colostomy. Dis Colon Rectum 2000;43:650-5

(6) Williams J. Sexual health: case study of a patient who has undergone stoma formation. Br J Nurs 2006;15:760-3

(7) Ma N, Harvey J, Stewart J, Andrews L, Hill AG. The effect of age on the quality of life of patients living with stomas: a pilot study. ANZ J Surg 2007;77:883-5

(8) B Holzer, Matzel K, Schiedeck T, Christiansen J, Christensen P, J Rius et al. Do geographic and educational factors influence the quality of life in rectal cancer patients with a permanent colostomy? Dis Colon Rectum 2005;48:2209-16

(9) Hamashima C. Long-term quality of life of postoperative rectal cancer patients. J Gastroenterol Hepatol 2002;17:571-6

(10) O'Connor G. Discharge planning in rehabilitation following surgery for a stoma. Br J Nurs 2003;12:800-7

(11) Williams J. Stoma care nursing: what the community nurse needs to know. Br J Community Nurs 2007;12:342-6

(12) Perrin A. Know how. Going home after stoma surgery. Community Nurse November 1999;5:26-7

(13) Turnbull GB, Erwin - Toth P. Ostomy care: foundation for teaching and practice. Ostomy Wound Manage 1999;45:23S-30S

(14) Pellino T, Tluczek A, Collins M, Trimborn S, Norwick H, Engelke ZK et al. Increasing self-efficacy through empowerment: preoperative education for orthopedic patients. Orthop Nurs 1998;17:48-9

(15) Ringhofer J. Meeting the needs of your ostomy patient. RN 2005;68:37-41

(16) Metcalf C. Stoma care: empowering patients through teaching practical skills. Br J Nurs 1999;8:593-600

(17) Doughty D. Role of the enterostomal therapy nurse in ostomy patient rehabilitation. Cancer 1992;70:1390-2

(18) Karadag A, Mentes BB, Uner A, Irkorucu O, Ayaz S, Ozkan S. Impact of stomatherapy on quality of life in patients with permanent colostomies or ileostomies. Int J Colorectal Dis 2003;18:234-8

(19) Chaudhri, S, Brown L, Hassan I, Horgan A. Preoperative intensive, community-based vs. traditional stoma education: a randomized, controlled trial. Dis colon rectum. 2005;48:504-9

(20) Colwell JC, Gray M. Does preoperative teaching and stoma site marking affect surgical outcomes in patients undergoing ostomy surgery? J Wound Ostomy Continence Nurs 2007;34:492-6
(21) Marquis P , Marrel A, Jambon B. Quality of life in patients with stomas: the Montreux Study. Ostomy Wound Manage 2003;49:48-55
(22) Jeter KF. Perioperative teaching and counseling. Cancer 1992;70:1346-9
(23) Borwell B. Continuity of care for the stoma patient: psychological considerations. Br J Community Nurs 2009;14:326,328,330-26,328,331
(24) Suhonen R, Valimaki M, Leino-Kilpi H. Individualized care, quality of life and satisfaction with nursing care. J Adv Nurs 2005;50:283-92
(25) Johansson K, Salanter S, Heikkinen K, Kuusisto A, Virtanen H, Leino-Kilpi H. Surgical Patient education: Assessing the intervention and exploring the outcomes from experimental and quasi-experimental studies from 1990 to 2003. Clinical Effectiveness in Nursing 2004;8:81-92
(26) R Suhonen , Virtanen H , Heikkinen, K , Johansson K , Kaljonen A Leppanen T et al . Health -related quality of life of day-case surgery patients: a pre/post-test survey using EuroQoL-5D. Qual Life Res 2008;17:169-77
(27) Johansson K, Leino-Kilpi H, Salantera S, Lehtikunnas H, Ahonen P, Elomaa E et al. Need for change in patient education: a Finnish survey from the patient's perspective. Patient Educ Couns 2003;51:239-45
(28) Hørdam B , Sabroe S, Pedersen PU, Mejdahl S, Søballe K. Nursing intervention by telephone interviews of patients aged over 65 years after total hip replacement improves health status: a randomized clinical trial. Scand J Car Sc. 2010;24:94-100.
(29) Grönvold M. [Health -related quality of life in cancer patients]. Ugeskr Laeger 2008;170:838-43
(30) Jess P. [Quality of life assessments in colorectal surgery]. Ugeskr Laeger 2008;170:853-5
(31) Burckhardt CS, Hanestad BR. Nursing strategies and quality of life outcomes: a systematic review. Nordic Journal of Nursing Research & Clinical Studies. 2003;23:4-9
(32) Wittrup-Jensen KU, Lauridsen JT, Moller Pedersen K. [Do Danes get the most for their money when prioritizing?].In: Lauridsen JT, Moller Pedersen K, editors. [Health. From Theory to Practice]. DJØF´s forlag.2009 p 161-74.
(33) van den Hout WB, van den Brink M, Stiggelbout AM, van de Velde CJ, Kievit J. Cost-effectiveness analysis of colorectal cancer treatments. Eur J Cancer 2002;38:953-63
(34) Cheung, K, Oemar M, Up M, Rabin R. EQ -5D User Guide. Basic information on how to-use-Eq-5D. 2009. Report Version 2.0
(35) Sorensen J, Davidsen M, Gudex C , Pedersen KM, Bronnum-Hansen H. Danish EQ-5D population norms. Scand J Public Health 2009;37:467-74
